# Seroprevalence of *Trypanosoma cruzi* infection in blood donors in the extreme South of Brazil

**DOI:** 10.1590/0037-8682-0599-2021

**Published:** 2022-04-29

**Authors:** Tanise Freitas Bianchi, Ana Paula da Paz Grala, Ítalo Ferreira de Leon, Sabrina Jeske, Gisele Ortiz Heidrich Pinto, Marcos Marreiro Villela

**Affiliations:** 1 Universidade Federal de Pelotas, Instituto de Biologia, Programa de Pós-Graduação em Parasitologia, Pelotas, RS, Brasil.; 2 Hemocentro Regional de Pelotas, Pelotas, RS, Brasil

**Keywords:** Chagas disease, Trypanosoma cruzi, Donors

## Abstract

**Background:**

We aimed to evaluate the seroprevalence of Chagas disease among blood donors in southern Rio Grande do Sul, Brazil.

**Methods:**

The study was conducted from 2010 to 2019 based on data registered by the Hemocentro Regional de Pelotas, Rio Grande do Sul.

**Results:**

There were 106,320 blood donations, and the discard rate of blood bags, either due to positive reactions to anti-*T. cruzi* antibodies or inconclusive results was 0.27% (283 bags).

**Conclusions:**

The usage of methods that enable the safe identification of donors with positive serology for Chagas disease is fundamental to ensure transfusional safety.

Chagas disease (CD), caused by the protozoan *Trypanosoma cruzi*, is considered a public health challenge in Latin America^1^. The Pan American Health Organization (PAHO) estimates that *T. cruzi* infects 6-8 million people, and approximately 10 thousand people die of CD in Latin America every year^2^.

The mean prevalence of CD in blood donors in Latin America was 0.2%^2^. The estimated prevalence of CD due to blood transfusion in Latin America was 1.3% in 2006, whereas in Brazil it was 0.21%, which showed a significant decrease as compared to the percentages found in previous years^1^_._

Rio Grande do Sul (RS) in southern Brazil is considered endemic to CD. *Triatoma infestans*, a species that is better adapted to households, is regarded as the main vector of CD. However, even though this vector was eliminated from southern RS, other species of triatomines have persisted in rural houses^3^ ^,^ ^4^ ^,^ ^5^.

A serological survey carried out by Camargo et al.^6^ showed that RS, along with Minas Gerais (MG), was the Brazilian state with the highest rate of human seroprevalence for *T. cruzi*, that is, 8.8% positivity among rural residents. A study conducted by Araújo et al.^7^ in southern RS found that the seroprevalence of *T. cruzi* infection was 2.7%.

Therefore, it is important to evaluate CD seroprevalence among blood donors in southern RS, Brazil. Thus, new data to update knowledge on the discarding of blood bags due to infections caused by *T*. *cruzi* in blood donor centers located in the region is relevant.

The study area stretched over cities that belong to the 3rd Coordenadoria Regional de Saúde (CRS) and 7th CRS. Both were located in the extreme south of Brazil. 

The 3rd CRS had its headquarters in Pelotas and encompassed 22 cities (Amaral Ferrador, Arroio do Padre, Arroio Grande, Capão do Leão, Canguçu, Cerrito, Chuí, Cristal, Herval, Jaguarão, Morro Redondo, Pedras Altas, Pedro Osório, Pelotas, Pinheiro Machado, Piratini, Rio Grande, Santa Vitória do Palmar, Santana da Boa Vista, São José do Norte, São Lourenço do Sul, and Turuçu). The 7th CRS was headquartered in Bagé and encompassed six cities (Aceguá, Bagé, Candiota, Dom Pedrito, Hulha Negra, and Lavras do Sul).

To evaluate the prevalence of anti-*T. cruzi* IgG antibodies among blood donors in southern Brazil, a study was conducted at the Hemocentro Regional de Pelotas (HEMOPEL), a blood donor center located in Pelotas, RS, which is responsible for collecting, fractionating, and distributing blood to all transfusion agencies that belong to the 3rd and the 7th CRS.

This retrospective and descriptive study was based on secondary data registered with the system in the 2010s. The results were based on the serological screening at HEMOPEL, as either negative soropositivy or inconclusive results for CD. The other variables were sex, age, schooling, occupation, birthplace, place of residence, and the following co-infections: hepatitis B (anti-HBC and HBsAg), hepatitis C (anti-HCV), HIV-1 and HIV-2 (anti-HIV and HIV Ag\Ac), syphilis (VDRL), and human lymphotropic virus types I and II (anti-HTLV-I and HTLV-II).

HEMOPEL was used to carry out the serological screening for CD using enzyme-linked immunosorbent assay (ELISA) up to 2015. However, chemiluminescent immunoassays (CLIA) has been regularly used to detect anti-*T. cruzi* IgG antibodies since then.

Regardless of whether the result of the assay was seropositive or inconclusive for infections caused by *T. cruzi*, the blood donors were called to undergo a second serological screening by CLIA. In both cases, blood bags were discarded. Therefore, the presence of positive and inconclusive results for the anti-*T*. *cruzi* antibodies in the second assay made the blood unsuitable for donation (discarded bags). Patients who tested seropositive were referred for medical appointments for more detailed examinations to determine whether their infections were recent or late and whether there were cardiac or digestive alterations associated with *T. cruzi*.

The project was approved by the Ethics Committee (no. 3,277,448). 

Data tabulation was conducted using Microsoft Excel®, which provided a database. Values are expressed as frequencies (observed value, n) and percentages. In addition to the variables under investigation, this study was divided into two quinquennia (2010-2014 and 2015-2019). The chi-square test was used for the statistical comparison of variables, and the significance level was set at 0.05. The MINITAB® 18 program was used for the statistical significance tests.

A total of three hundred and twenty blood donations were registered at HEMOPEL between 2010 and 2019. A total of 102 donors tested seropositive for anti-*T. cruzi* IgG antibodies (either the first and second samples tested positive or the first test was inconclusive and the second tested positive, whereas the blood samples from 181 donors showed inconclusive results for anti-*T. cruzi* IgG antibodies (first and second samples gave inconclusive results). Thus, 283 (0.27%) blood bags were unsuitable for donation because of CD confirmation or suspicion (Table 1). 

The results showed a statistically significant difference in the positive test results of blood donors for anti-*T. cruzi* IgG antibodies between the quinquennia (2010-2014 and 2015-2019). There were decreases in seropositive donors and inconclusive results in the second quinquennium (p <0,01) (Table 1). 

**TABLE 1:** Distribution of the blood samples screened for anti-*T. cruzi* IgG in donors from a blood center in the extreme south of Brazil from 2010 to 2019.

Year*	Screened blood samples	**Reactive blood samples for anti*-T. cruzi***	**Blood samples inconclusive for anti*-T. cruzi* antibodies**	**Disposal of blood bags due to *T. cruzi* positivity (or suspicion)**
	(n)	n (%)	n (%)	n*(%)
2010^a^	8,389	11 (0.13)	16 (0.19)	27 (0.32)
2011^a^	8,123	15 (0.18)	14 (0.17)	29 (0.35)
2012 ^a^	8,800	11 (0.12)	9 (0.10)	20 (0.22)
2013 ^a^	10,576	4 (0.03)	32 (0.30)	36 (0.34)
2014 ^a^	12,834	11 (0.08)	30 (0.23)	41 (0.31)
2015 ^b^	11,518	17 (0.14)	45 (0.39)	62 (0.53)
2016 ^b^	10,832	14 (0.12)	7 (0.06)	21 (0.19)
2017 ^b^	11,538	3 (0.02)	9 (0.07)	12 (0.10)
2018 ^b^	11,826	5 (0.04)	10 (0.08)	15 (0.12)
2019 ^b^	11,884	11 (0.09)	9 (0.07)	20 (0.16)
**Total**	**106,320**	102(0.09)	**181(0.17)**	**283 (0.26)**

^a^ ^b^ Comparing the five years 2010-4; 2015-9; p <0,01.

The analysis of the profiles of blood donors who showed soropositive for DC showed that most were single 46-60-year-old men with low schooling. There was a statistical association between CD and blood donor age, that is, individuals who were ≥46 years old exhibited positive or inconclusive results for anti-*T. cruzi* IgG antibodies significantly more frequently (p <0,01) (Table 2).

**TABLE 2:** Profile of blood donors with positive and inconclusive results for anti-T. *cruzi* from a blood center in the extreme south of Brazil from 2010 to 2019.

Variables	**Reactive serology for *T. cruzi***	**Serology inconclusive for *T. cruzi***	Total
Age group*			
18-30	10 (3.53)	57 (20.14)	67 (23.67)
31-45	22 (7.77)	51 (18.02)	73 (25.8)
46-60	47 (16.61)	40 (14.13)	87 (30.74)
>60	23 (8.13)	33(11.66)	56 (19.79)
**Gender**			
Female	47 (16.61)	70 (24.73)	117 (41.34)
Male	55 (19.43)	111 (39.22)	166 (58.66)
**Marital status**			
Married	39 (13.78)	54 (19.08)	93 (32.86)
Single	46 (16.25)	103 (36.40)	149 (52.65)
Divorced	10 (3.53)	12 (4.24)	22 (7.77)
Widowed	4 (1.41)	6 (2.12)	10 (3.53)
Others	3 (1.06)	6 (2.12)	9 (3.18)
**Educational level**			
Illiterate	2 (0.71)	-	2 (0.71)
Incomplete Elementary School	46(16.25)	48 (16.96)	94 (33.22)
Complete Elementary School	17 (6.01)	15 (5.30)	32 (11.31)
Incomplete high school	7(2.47)	8 (2.83)	15 (5.30)
Complete high school	19 (6.71)	37 (13.07)	56 (19.79)
Incomplete university	5 (1.77)	45 (15.90)	50 (17.67)
Graduated university	6 (2.12)	28 (9.89)	34 (12.01)

*Chi-square test performed for groups 18-45 / ≥46; p <0,01 (significant).

The city of residence that exhibited the highest number of donors who showed soropositive for DC *T. cruzi* was Pelotas (24.4%), followed by Santana da Boa Vista (3.5%) (Table 3). However, regarding blood donor birthplace, 26 (9.2%) individuals who were seropositive for anti-*T. cruzi* antibodies were born in Canguçu, whereas 25 (8.8%) were born in Pelotas.

**TABLE 3:** Current city of residence of blood donors with positive and inconclusive results for anti-*T. cruzi* from a blood center in the extreme south of Brazil from 2010 to 2019.

Current municipality	**Soropositive for *T.cruzi***	**Inconclusive for *T.cruzi* infection**	Total
Canguçu	7 (2.47)	2 (0.71)	9 (3.18)
Capão do Leão	6 (2.12)	9 (3.18)	15 (5.30)
Cerrito	2 (0.71)	2 (0.71)	4 (1.41)
Pelotas	69 (24.38)	138 (48.76)	207 (73.14)
Pinheiro Machado	2 (0.71)	1 (0.35)	3 (1.06)
Piratini	-	4 (1.41)	4 (1.41)
Rio Grande	-	7 (2.47)	7 (2.47)
Santana Boa Vista	10 (3.53)	3 (1.06)	13 (4.59)
Others	6 (2.12)	15 (4.95)	21 (7.42)
**Total**	**102 (36)**	**181 (64)**	**283 (100)**

Serological markers were also evaluated in those seropositive for CD. Among the seropositive donors, 0.71% (2) showed positivity for hepatitis B serological markers.

The discard rate of blood bags owing to either positive or inconclusive reactions to anti-*T. cruzi* IgG antibodies at HEMOPEL in the 10 years was 0.27% (283 blood bags). This result was 80% higher than the general prevalence rate of seroreactivity to *T. cruzi* among candidates for blood donation in Brazil (0.15 %). RS exhibited 0.12%, as shown by data collected in 2019 and issued by ANVISA^8^. Even so, the result found in this study is close to the mean prevalence in blood donors in Latin America (0.2%)^2^.

A study of blood donors carried out by Araújo et al.^7^ in Pelotas, RS, showed high seropositivity to *T. cruzi* (0.98 %). The difference may be because positive or inconclusive serology for CD has gradually decreased over the years. This was observed by this study based on the statistically significant difference between the quinquennia under evaluation. However, the differences in the techniques used for the serological diagnosis of CD may also be responsible for such events. 

Pedroso et al.^9^ carried out a study in which the seroprevalence values were much higher than those of this study; they found that 2.7% of donations were seroreactive to *T. cruzi* in northwestern RS, where CD is considered the leading cause of blood bag discard among the serologically assayed infectious diseases. The northwestern region in RS was the last one that eliminated the main vector of *T*. *cruzi*, *Triatoma infestans*, since it had residual foci of the species up to 2014^3^. Thus, vector transmission in the recent decades may explain the high seropositivity shown by blood banks in that region. 

Most ineligible donors were 46-60 years old (30.74%). These data corroborate the findings of Lopes et al.,^10^ who showed a positive correlation between an increase in age and the percentage of patients who were seropositive for CD. Moraes-Souza et al.^11^ pointed out that 70% of ineligible donors were over 30 years old. In both cases, the authors related the high positivity in older patients to high rates of triatomine infestation and colonization found in rural houses in the past. 

Individuals from Canguçu and Pelotas formed the largest group of donors who were seroreactive to anti-*T. cruzi* IgG antibodies. Fitarelli and Horn^12^ (2009) corroborated these findings by showing that the discard rate of blood bags due to CD at hospitals in Canguçu was approximately 2-fold of that found in other places under investigation. In addition, Baruffa and Alcântara^13^ found the most prominent focus of *T*. *infestans* in houses in Canguçu and Piratini, where several cases of acute CD were registered. The study conducted by Bianchi et al.^4^ also corroborates these results since they noted the largest number of captured triatomines in Canguçu, where 37.7% of all insects, mainly *Triatoma rubrovaria,* captured in 22 southern Brazilian cities were found. 

Serological markers were also evaluated in those seropositive for CD. No cases of donors coinfected with hepatitis C, syphilis, HTLV I and II, and HIV were found in this investigation. Regarding *T. cruzi*/HIV coinfection, a negative result requires attention because the prevalence of coinfected patients in the study area was 3.8-fold higher than that estimated by the Ministry of Health, as reported by Staufertt et al.^14^. However, this investigation was conducted in a specialized care facility for HIV-positive patients. Regarding hepatitis B serological markers, only two donors (0.71 %) were positive for anti-*T. cruzi* antibodies and co-infections tended to worsen an individual’s clinical condition; thus, it is fundamental to forward these cases to specialized care facilities^15^. 

Although the discard rate of blood bags owing to serologically positive and inconclusive reactions to anti-*T. cruzi* IgG antibodies is relatively low (0.27% in approximately 100 thousand samples under analysis), it is essential to carry out serological assays to avoid transfusional CD infections. The use of molecular techniques may help reduce the number of inconclusive cases. In addition, certain cities in the extreme south of Brazil, mainly Canguçu, are prone to have patients seropositive to CD. These data can be used to develop further detailed studies in this region. Candidates for blood donation who are soropositive must receive adequate medical treatment and social care.

## References

[1] Dias JCP, Ramos AN, Gontijo ED, Luquetti A, Shikanai-Yasuda MA, Coura JR (2016). II Consenso Brasileiro em doença de Chagas, 2015. Epidemol Serv Saude.

[2] OPAS.Organização Pan-Americana da Saúde (2021). OPAS: 70% das pessoas com Chagas não sabem que estão infectadas.

[3] Bedin C, Wilhelms T, Villela MM, Silva GCCD, Riffel APK, Sackis P (2021). Residual foci of Triatoma infestans infestation: Surveillance and control in Rio Grande do Sul, Brazil, 2001-2018. Rev Soc Bras Med Trop.

[4] Bianchi TF, Jeske S, Grala APDP, Leon IFD, Bedin C, Mello FD (2021). Current situation of Chagas disease vectors (Hemiptera, Reduviidae) in Southern Rio Grande do Sul State, Brazil. Rev Inst Med Trop Sao Paulo.

[5] Priotto M, Dos Santos CV, De Mello F, Ferraz ML, Villela MM (2014). Aspectos da vigilância entomológica da doença de Chagas no sul do Rio Grande do Sul, Brasil. Rev Patol Trop.

[6] Camargo ME, Silva GR, Castilho EA, Silveira AC (1984). Inquérito sorológico de prevalência da infecção chagásica no Brasil. 1975/1980. Rev Inst Med Trop.

[7] Araújo AB, Vianna EES, Berne MEA (2008). Anti-Trypanosoma cruzi antibody detection in blood donors in the Southern Brazil. Braz J Infect Dis.

[8] Agência Nacional de Vigilância Sanitária (ANVISA) (2021). 8º Boletim de Produção Hemoterápica do Brasil.

[9] Pedroso D, Santos CV, Novicki A, Berne MEA, Villela MM (2016). Estudo retrospectivo de sororreatividade para Trypanosoma cruzi em doadores de sangue da região noroeste do Rio Grande do Sul, Brasil. Rev Patol Trop.

[10] Lopes PS, Ramos ELP, Gómez-Hernández C, Ferreira GLS, Rezende-Oliveira K (2015). Prevalence of Chagas disease among blood donor candidates in Triangulo Mineiro, Minas Gerais state, Brazil. Rev Inst Med Trop Sao Paulo.

[11] Moraes-Souza H, Martins PRJ, Pereira GA, Ferreira-Silva MM, Abud MB (2006). Serological profile concerning Chagas’ disease of blood donors at Uberaba Blood Center. Rev Bras Hematol Hemoter.

[12] Fitarelli DB, Horn JF (2009). Disposal of blood units due to reactivity for Chagas’ disease in a blood donor serological screening laboratory in Porto Alegre, Brazil. Rev Bras Hematol Hemoter.

[13] Baruffa G, Fo Alcantara A (1985). Inquérito sorológico e entomológico da infecção pelo T. cruzi na região Sul do Rio Grande do Sul, Brasil. Ann Soc Belg Med Trop.

[14] Stauffert D, Silveira MF, Mesenburg MA, Manta AB, Dutra AS, Bicca GL (2017). Prevalence of Trypanosoma cruzi/HIV coinfection in southern Brazil. Braz J Infect Dis.

[15] da Rocha LB, Mariño JM, da Silva Reis MH, Portugal JKA, da Silva Barão ÉJ, de Freitas DLA (2020). Soroprevalência de doenças infecciosas em doadores de sangue em um município do Amazonas. REAS.

